# Stay or Go: Sulfolobales Biofilm Dispersal Is Dependent on a Bifunctional VapB Antitoxin

**DOI:** 10.1128/mbio.00053-23

**Published:** 2023-04-10

**Authors:** April M. Lewis, Daniel J. Willard, Mohamad J. H. Manesh, Shamphavi Sivabalasarma, Sonja-Verena Albers, Robert M. Kelly

**Affiliations:** a Department of Chemical and Biomolecular Engineering, North Carolina State University, Raleigh, North Carolina, USA; b Institute for Biology, Molecular Biology of Archaea, University of Freiburg, Freiburg, Germany; c Spemann Graduate School of Biology and Medicine, University of Freiburg, Freiburg, Germany; d Signalling Research Centres BIOSS and CIBBS, Faculty of Biology, University of Freiburg, Freiburg, Germany; Binghamton University; Korea Advanced Institute of Science and Technology

**Keywords:** toxin-antitoxin loci, biofilms, Sulfolobales, thermoacidophiles, *Sulfolobus acidocaldarius*

## Abstract

A type II VapB14 antitoxin regulates biofilm dispersal in the archaeal thermoacidophile Sulfolobus acidocaldarius through traditional toxin neutralization but also through noncanonical transcriptional regulation. Type II VapC toxins are ribonucleases that are neutralized by their proteinaceous cognate type II VapB antitoxin. VapB antitoxins have a flexible tail at their C terminus that covers the toxin’s active site, neutralizing its activity. VapB antitoxins also have a DNA-binding domain at their N terminus that allows them to autorepress not only their own promoters but also distal targets. VapB14 antitoxin gene deletion in S. acidocaldarius stunted biofilm and planktonic growth and increased motility structures (archaella). Conversely, planktonic cells were devoid of archaella in the Δ*vapC14* cognate toxin mutant. VapB14 is highly conserved at both the nucleotide and amino acid levels across the Sulfolobales, extremely unusual for type II antitoxins, which are typically acquired through horizontal gene transfer. Furthermore, homologs of VapB14 are found across the *Crenarchaeota*, in some *Euryarchaeota*, and even bacteria. S. acidocaldarius *vapB14* and its homolog in the thermoacidophile Metallosphaera sedula (Msed_0871) were both upregulated in biofilm cells, supporting the role of the antitoxin in biofilm regulation. In several Sulfolobales species, including *M. sedula,* homologs of *vapB14* and *vapC14* are not colocalized. Strikingly, Sulfuracidifex tepidarius has an unpaired VapB14 homolog and lacks a cognate VapC14, illustrating the toxin-independent conservation of the VapB14 antitoxin. The findings here suggest that a stand-alone VapB-type antitoxin was the product of selective evolutionary pressure to influence biofilm formation in these archaea, a vital microbial community behavior.

## INTRODUCTION

Billions of years ago, prokaryotic organisms developed the ability to form biofilms (1), largely beneficial multicellular communities that confer resistance to stressors and aid in nutrient sequestration. Archaeal biofilms have been studied most intently in the Sulfolobales, particularly Sulfolobus acidocaldarius, Saccharolobus solfataricus (f. Sulfolobus solfataricus), and Sulfurisphaera tokodaii (f. Sulfolobus tokodaii), which have various abilities to form biofilms despite similar extracellular polysaccharide matrix makeups (2). Some thermoacidophiles can form biofilms on sulfur (Sulfuracidifex metallicus [f. Sulfolobus metallicus]) (3) and iron pyrite (*Sulfu. metallicus* and *Acidianus* spp.) (4), substrates that serve as energy sources. There are three major phases of biofilm formation: attachment, maturation, and dispersal. In archaeal biofilms, attachment is typically mediated by type IV pili, proteinaceous structures that irreversibly adhere to surfaces and attach to other cells (5). S. acidocaldarius has three different type IV pili or type IV pilus-like appendages: the archaeal adhesive pili (Aap) (6), the situational UV-inducible pili (Ups) (7), and the primary motility appendage (the archaeal flagellum), referred to as the archaellum (6, 8). S. acidocaldarius primarily attaches to surfaces by the Aap pilus, although removal of the UV-inducible pili also has an acute effect on biofilm morphology (6). During maturation, attached cells produce an extracellular matrix consisting of polysaccharides, protein, lipids, and extracellular DNA (eDNA) (9). S. acidocaldarius, *Sa. solfataricus*, and *Sulfu. tokodaii* have extracellular matrices composed of polysaccharides containing glucose, galactose, mannose, and *N*-acetylglucosamine (2, 10), with protein structures and some eDNA also detected in S. acidocaldarius biofilms (11). Archaeal biofilm dispersal is mediated by the archaellum (6, 8), which propels cells to new locations potentially to form biofilm through reversible interactions with the substratum. In fact, deletion of the archaellum in S. acidocaldarius (6) leads to attachment defects.

Control of biofilm formation in S. acidocaldarius is accomplished by several known regulators. The one-component system ArnR (archaellar regulatory network regulator) and its homolog, ArnR1, both act as activators of archaella by directly binding to the *arlB* gene promoter during nitrogen starvation (12). However, only ArnR binds to the promoter regions and directly induces expression of both *aapF* and *upsX* pilus genes (12, 13). Additionally, the leucine-responsive regulator 14 (Lrs14)-like proteins of S. acidocaldarius, encoded by Saci_1223 and Saci_1242, are biofilm activators and result in an impaired biofilm upon deletion (14). Moreover, archaeal biofilm regulator 1 (AbfR1), another Lrs14-like regulator, is a known repressor of biofilm formation that, depending on its phosphorylation state, directly binds to *aap* and *arl* (archaellum genes) promoters (15). Deletion mutants of *abfR1* downregulate *arl* genes, leading to decreased motility, and upregulate *aap* genes, causing increased biofilm formation (14).

Beyond these mechanisms, other factors that regulate this phenomenon remain unknown. In mesophilic bacteria, toxin-antitoxin (TA) loci have been connected to biofilm formation. Type II TAs are most prevalent and consist of a toxin protein that typically functions as a RNase and an antitoxin protein (16). The antitoxin possesses a C terminus that obstructs the toxin active site, neutralizing its RNase activity, and a DNA-binding N terminus that autoregulates the TA operon (16–20). In several mesophilic bacteria, perturbing native TA systems leads to defects in biofilm formation. Deletion of TA systems in Escherichia coli caused defects in quorum sensing and biofilm attachment (21, 22), and stunted biofilm formation was observed in TA mutants of Vibrio cholerae (23) and Streptococcus pneumoniae (24). In Staphylococcus aureus, deletion of the *mazF* type II toxin gene also caused increased biofilm formation and higher sensitivity to antibiotic treatment (25). In Caulobacter crescentus, a ParDE_4_ TA system is activated upon O_2_ limitation and enhances eDNA-stimulated dispersal from its biofilm, perhaps to seek out a more favorable situation (26). Interestingly, the antitoxin’s DNA-binding domain not only binds to its own promoter region, particularly when complexed with its cognate toxin (27, 28), but also can bind to and regulate distal promoters (22, 29, 30). For example, the E. coli type II antitoxin MqsA represses its own operon and that of the noncognate *cspD* toxin (22). Additionally, MqsA represses the *rpoS* stress response sigma factor, which reduces the level of cyclic-di-GMP, causing an increase in motility and decrease in biofilm (29). Moreover, all mutants of the Pseudomonas putida *mqsRA* toxin-antitoxin locus showed significant biofilm defects. Not only did TA systems promote P. putida biofilm formation, but the antitoxin MqsA repressed a sigma factor and a universal stress protein (30), illustrating the antitoxin’s dual role of toxin neutralizer and transcriptional regulator.

Much less is known about the function of TA systems in the *Archaea*. Studies have shown that TA systems play a role in the heat shock response of *Sa. solfataricus* (31, 32) and the response of *Metallosphaera* spp. to uranium exposure (33, 34). TA systems were transcriptionally upregulated in the thermoacidophile *Sa. solfataricus* during heat shock, and deletion of the *vapB6* antitoxin gene resulted in a heat-labile mutant (31). Furthermore, Metallosphaera prunae developed resistance to hexavalent uranium—more so than its close relative Metallosphaera sedula—by degrading its own cellular RNA via toxin ribonucleases, resulting in growth arrest and entry into a dormant state (33, 34).

Currently, the toxin RNase component of type II TA systems has attracted the most attention. Rarely have separate antitoxin mutants been examined and in fewer cases has the regulatory ability of the antitoxin been investigated outside its own operon. Furthermore, toxin-antitoxin systems are often acquired through horizontal gene transfer, leading to a lack of nucleotide identity conservation, even within a species (35). Here, we focus on an archaeal type II antitoxin’s regulatory function beyond its own promoter, in which a VapB antitoxin had a profound effect on biofilm formation in S. acidocaldarius. The nucleotide identity of this antitoxin, VapB14, was highly conserved across the Sulfolobales and responded similarly to biofilm growth in *M. sedula*, suggesting a significant role in regulating this phenomenon across the Sulfolobales.

## RESULTS

### Sulfolobus acidocaldarius biofilm transcriptome.

**FIG 1:**
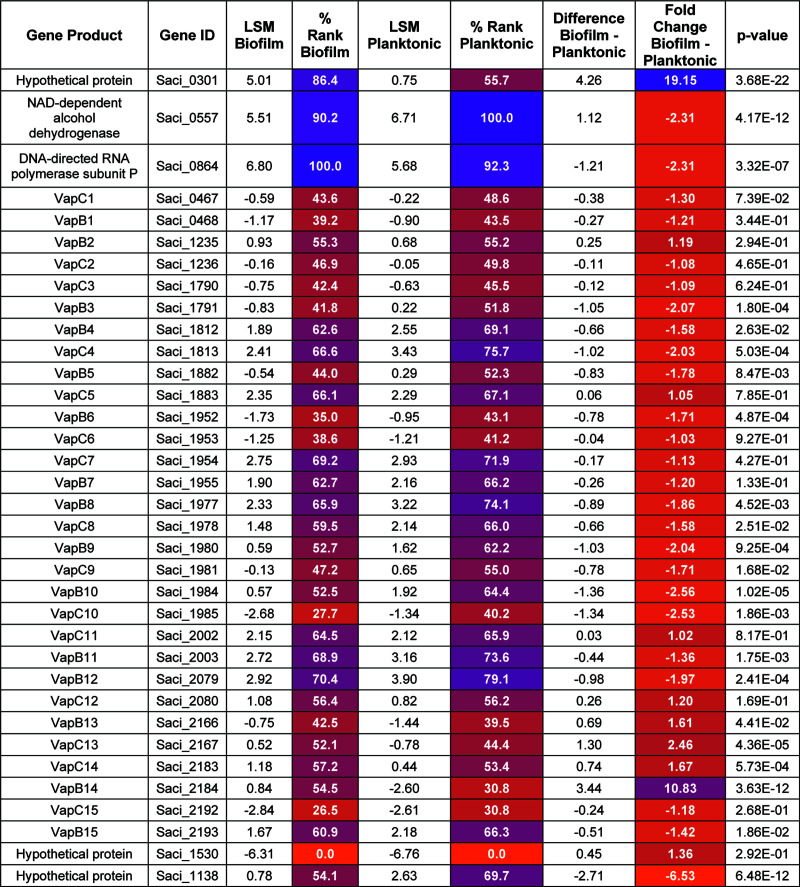
Sulfolobus acidocaldarius *vapBC* transcriptional response to biofilm formation. Shown are microarray data measuring differential expression of genes between biofilm and planktonic Sulfolobus acidocaldarius MW001 cells.

**FIG 2:**
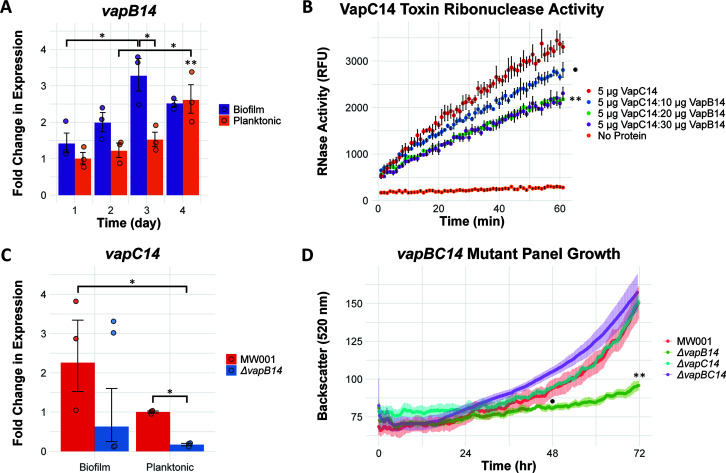
S. acidocaldarius VapBC14 expression and activity. (A) Differential *vapB14* expression in S. acidocaldarius biofilm and planktonic cultures. Shown are results from relative qPCR of total RNA isolated from S. acidocaldarius MW001 biofilm and planktonic cultures across 4 days of growth. Graphed data represent the average fold change of *vapB14* expression of *n* = 3 biological replicates compared to the day 1 planktonic condition ± standard error of the mean (SEM). Graphed points are the fold change in individual biological replicates. Asterisks indicate statistically significant difference compared to the day 1 planktonic condition: *, *P* < 0.05; **, *P* < 0.01. (B) Reduction of VapC14 RNase activity by VapB14 antitoxin. *n* = 3 experimental replicates ± SEM. Statistically significant difference from 5 μg VapC14 toxin alone at 60 min: **, *P* < 0.01; ●, *P* < 0.10. (C) Differential *vapC14* expression in S. acidocaldarius MW001 and Δ*vapB14* mutant biofilm and planktonic cultures. Shown are results from relative qPCR of total RNA isolated from S. acidocaldarius MW001 and Δ*vapB14* mutant biofilm and planktonic cultures after 3 days of growth. Graphed data represent the average fold change of *vapC14* expression of *n* = 3 biological replicates (*n* = 4 for Δ*vapB14* mutant biofilm) compared to the MW001 parent planktonic condition ± SEM. Graphed points are the fold change in individual biological replicates. *, *P* < 0.05. (D) S. acidocaldarius MW001 parent and Δ*vapB14*, Δ*vapC14*, and Δ*vapBC14* mutant planktonic growth curves monitored by back scatter at 520 nm. Each curve represents the average of *n* = 4 independent experiments ± SEM. The Δ*vapB14* mutant is significantly different from the MW001 parent at 48 h (●, *P* < 0.10) and 72 h (**, *P* < 0.05). Statistical differences were calculated using a two-way analysis of variance (ANOVA) followed by a Tukey honestly significant difference (HSD) *post hoc* test for panels A, B, and D and a Dunnett T3 test for panel C.

Transcriptomics comparing biofilm to planktonic S. acidocaldarius cells identified genes associated with biofilm formation. Among the most biofilm-responsive genes was Saci_2184, expression of which increased 10.8-fold in the biofilm compared to planktonic culture, second only to the hypothetical protein gene Saci_0301 (Fig. 1). The high transcriptional response of Saci_0301 was previously reported, and its deletion causes a biofilm defect, which was found to be regulated by a noncoding RNA, RrrR (RNase R-resistant RNA) (36). Saci_2184 and Saci_2183 encode a putative VapB type II antitoxin and its cognate VapC toxin, respectively. Using TAfinder (37), 17 type II toxin-antitoxin pairs are predicted in the S. acidocaldarius genome (Table 1; see Fig. S1 in the supplemental material). The clustering of TA loci in the genome indicates a possible horizontal inheritance by mobile genetic elements (Fig. S1). Of these 17 potential TA pairs, two sets, Saci_1957/Saci_1956 and Saci_2111/Saci_2112, are not associated with any TA type and have predicted toxins of an abnormally long length, suggesting they are unlikely to be type II TAs. One identified TA pair was predicted as an MNT/HEPN-like system, which is common to thermophiles (38). MNT (minimal nucleotidyltransferase)-type antitoxins inactivate their cognate HEPN (higher eukaryotes and prokaryotes nucleotide-binding) ribonucleases by AMPylation (39). The remaining TAs were identified as the VapBC type, including Saci_2184 (referred to here as VapB14). Interestingly, the VapB14 predicted cognate VapC-type toxin Saci_2183 (referred to here as VapC14) was unresponsive in biofilm cells. In fact, except for *vapB14*, all other identified toxin or antitoxin genes were largely unresponsive in the biofilm (Fig. 1). All toxin and antitoxin genes, including *vapB14*, were between the 25th and 75th percentiles of the transcriptome profile for both the biofilm and planktonic conditions. Follow-up relative quantitative real-time PCR (qPCR) analysis of S. acidocaldarius MW001 biofilm and planktonic cultures revealed a significant 2.2-fold increase in *vapB14* expression under the biofilm condition on day 3 (Fig. 2A), confirming these results. Moreover, *vapB14* is upregulated 2.9-fold in Saci_1223 (biofilm activator) mutant planktonic cells compared to the parent, suggesting that Saci_1223 is a repressor of *vapB14* in planktonic growth (Table 2).

**TABLE 1:** Sulfolobus acidocaldarius TAfinder results^*a*^

Name	ID	T/A^*b*^	Locus tag	Location	Length (aa)	Strand	Family	Domain
VapC1	70606299	T	Saci_0467	394110–394478	122	−	PIN-like	cd09981
VapB1	70606300	A	Saci_0468	394465–394713	82	−	RHH-like	COG1753
VapC2	70607005	T	Saci_1236	1052274–1052666	130	+	VapC	
VapB2	70607004	A	Saci_1235	1052045–1052290	81	+	VapB	
VapC3	70607519	T	Saci_1790	1557436–1557846	136	−	PIN-like	COG4113
VapB3	70607520	A	Saci_1791	1557834–1558100	88	−	RHH-like	PRK11235
VapC4	70607542	T	Saci_1813	1580918–1581340	140	+	PIN-like	
VapB4	70607541	A	Saci_1812	1580688–1580921	77	+	RHH-like	
VapC5	70607611	T	Saci_1883	1677052–1677447	131	+	PIN-like	COG4113
VapB5	70607610	A	Saci_1882	1676838–1677062	74	+	RHH-like	COG3905
VapC6	70607679	T	Saci_1953	1765576–1765995	139	+	vapC	
VapB6	70607678	A	Saci_1952	1765335–1765583	82	+	vapB	
VapC7	70607680	T	Saci_1954	1766204–1766599	131	−	PIN-like	cd09872
VapB7	70607681	A	Saci_1955	1766583–1766810	75	−	AbrB-like	COG2002
NA	70607683	T	Saci_1957	1767340–1769043	567	−		pfam12568
NA	70607682	A	Saci_1956	1766910–1767353	147	−		COG1733
MNT8	70607703	T	Saci_1978	1792029–1792373	114	+	MNT-like	
HEPN8	70607702	A	Saci_1977	1791620–1792051	143	+	HEPN-like	
VapC9	70607706	T	Saci_1981	1795579–1795983	134	+	PIN-like	
VapB9	70607705	A	Saci_1980	1795356–1795592	78	+	RHH-like	
VapC10	70607710	T	Saci_1985	1799655–1800041	128	+	PIN-like	cd09886
VapB10	70607709	A	Saci_1984	1799411–1799665	84	+	AbrB-like	COG2002
VapC11	70607724	T	Saci_2002	1817055–1817375	106	−	PIN-like	
VapB11	70607725	A	Saci_2003	1817476–1817742	88	−	RHH-like	
VapC12	70607797	T	Saci_2080	1898348–1898716	122	+	PIN-like	COG4113
VapB12	70607796	A	Saci_2079	1898115–1898345	76	+	RHH-like	pfam01402
NA	70607828	T	Saci_2111	1932477–1934174	565	+		pfam12568
NA	70607829	A	Saci_2112	1934285–1934725	146	+		COG1733
VapC13	70607880	T	Saci_2167	2000715–2001335	206	+	VapC	
VapB13	70607879	A	Saci_2166	2000353–2000703	116	+	VapB	
VapC14	70607892	T	Saci_2183	2019340–2019957	205	−	VapC	
VapB14	70607893	A	Saci_2184	2019983–2020327	114	−	VapB	
VapC15	70607901	T	Saci_2192	2032246–2032686	146	−	PIN-like	
VapB15	70607902	A	Saci_2193	2032646–2032906	86	−	AbrB-like	

a TAfinder results generated using the Sulfolobus acidocaldarius DSM 639 complete genome (length = 2,225,959 bp).

b T/A indicates the type of the protein: T for toxin and A for antitoxin.

**TABLE 2:** Sulfolobus acidocaldarius *vapBC14* transcriptional response to Saci_1223 deletion^*a*^

Gene product	Gene ID	Differential expression							
		Biofilm vs planktonic				ΔSaci_1223 mutant vs MW001			
		MW001		ΔSaci_1223 mutant		Biofilm		Planktonic	
		Fold change	*P* value	Fold change	*P* value	Fold change	*P* value	Fold change	*P* value
VapC14 toxin	Saci_2183	1.7	5.73E−04	1.4	2.51E−02	1.4	1.28E−02	1.7	4.02E−04
VapB14 antitoxin	Saci_2184	10.8	3.63E−12	3.9	6.29E−07	1.0	8.62E−01	2.9	4.10E−05

a Shown are microarray data measuring differential expression of genes between biofilm and planktonic cells of the *S. acidocaldarius* MW001 parent and the ΔSaci_1223 mutant.

10.1128/mbio.00053-23.1 FIG S1 Location of identified type II toxin-antitoxin in the Sulfolobus acidocaldarius genome. Download FIG S1, TIF file, 14.3 MB. Copyright © 2023 Lewis et al. 2023 Lewis et al. https://creativecommons.org/licenses/by/4.0/ This content is distributed under the terms of the Creative Commons Attribution 4.0 International license.

### Reduction of VapC14 toxin activity by its cognate VapB14 antitoxin.

The VapC14 toxin and the VapB14 antitoxin were recombinantly expressed and tested for their associated activities. Purified fractions of the VapC14 toxin had a pinkish hue and correlated with the ~25-kDa VapC14 toxin band on an SDS-PAGE gel (Fig. S2). This coloration in VapC14-containing fractions may be due to the coelution of manganese ions, pinkish in aqueous solutions, that crystal structure studies indicate are present within VapC-type toxin active sites (17, 18). Additionally, significant RNase activity was measured in 5 μg of the VapC14 compared to the no-protein control, confirming its function as an RNase-type toxin (Fig. 2B; Fig. S3). No RNase activity was measured in the VapB14 antitoxin alone, and the VapC14 toxin’s activity was completely abolished by the addition of the metal ion-chelating agent EDTA (Fig. S3). Addition of 10 μg of the VapB14 antitoxin led to a mild 15% reduction in detectable VapC14 toxin activity. However, VapC14 RNase activity was significantly reduced with the addition of 20 μg (34%) and 30 μg (30%) of VapB14, confirming the antitoxin function of VapB14 (Fig. 2B).

10.1128/mbio.00053-23.2 FIG S2 Purification of the VapC14 toxin. (A) VapC14 toxin purification chromatogram from VapBC14 coexpression; (B) VapC14 toxin purification fractions’ coloration and gel. Download FIG S2, TIF file, 2.1 MB. Copyright © 2023 Lewis et al. 2023 Lewis et al. https://creativecommons.org/licenses/by/4.0/ This content is distributed under the terms of the Creative Commons Attribution 4.0 International license.

10.1128/mbio.00053-23.3 FIG S3 Ribonuclease activity of the VapC14 toxin reduction of VapC14 RNase activity by VapB14 antitoxin. *n* = 3 experimental replicates ± SEM. Statistically significant difference from the no-protein control at 60 min (**, *P* < 0.01) was calculated using a one-way analysis of variance (ANOVA) followed by a Tukey honestly significant difference (HSD) *post hoc* test. Download FIG S3, TIF file, 14.1 MB. Copyright © 2023 Lewis et al. 2023 Lewis et al. https://creativecommons.org/licenses/by/4.0/ This content is distributed under the terms of the Creative Commons Attribution 4.0 International license.

### Regulation of the *vapBC14* locus.

Type II toxin-antitoxin systems are typically organized in an operon with the antitoxin upstream and overlapping the toxin or separated by a small intergenic region (35). This paradigm also applies to the *vapBC14* locus as the *vapB14* antitoxin gene is upstream of the *vapC14* gene, with only a 25-bp intergenic region. Often type II antitoxins autorepress their own operon in conjunction with their cognate toxin through a process called conditional cooperativity; repression of the operon is dependent on the ratio of toxin to antitoxin in the regulating TA complex (27, 28). If the ratio is skewed toward antitoxin binding, repression occurs; if the ratio is skewed toward toxin, repression is relieved. If autorepression was the only impact on the expression of the *vapBC14* locus, then deletion of the *vapB14* antitoxin gene would cause an increase in the transcription of *vapC14.* However, qPCR showed a significant 5.7-fold decrease in *vapC14* expression in the Δ*vapB14* mutant compared to the MW001 parent strain in planktonic cultures (Fig. 2C), indicating the VapB14 antitoxin may not be regulating the *vapC14* promoter, as predicted.

### Role of the VapB14 antitoxin in planktonic and biofilm growth.

Single and double deletion mutants were generated for the *vapBC14* locus, and continuous monitoring of planktonic cultures was performed to determine culture fitness (Fig. 2D). The Δ*vapBC14* toxin-antitoxin mutant and the Δ*vapC14* toxin mutant grew similarly to the MW001 parent strain. However, planktonic growth of the Δ*vapB14* antitoxin mutant exhibited a significant growth defect compared to any other strain at 48 and 72 h (Fig. 2D): 14% and 36% less than MW001, respectively. Neither the Δ*vapC14* toxin nor the Δ*vapBC14* toxin-antitoxin mutants were significantly different from the parent at any time point. Additionally, *vapB14* expression increased a significant 2.6-fold in MW001 day 4 compared to day 1 planktonic cultures, indicating that this antitoxin may also play a role in late-stationary-phase growth (Fig. 2A).

**FIG 3:**
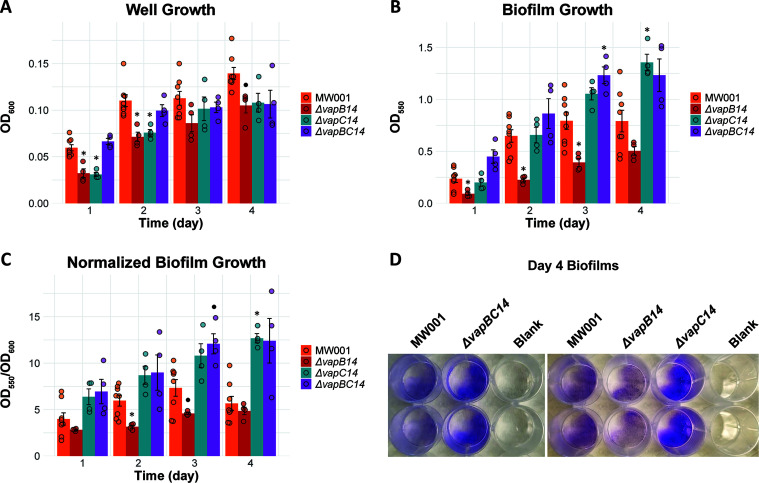
S. acidocaldarius *vapBC14* locus impact on biofilm and planktonic growth. (A to C) MW001 parent strain, Δ*vapB14* antitoxin mutant, Δ*vapC14* toxin mutant, and Δ*vapBC14* toxin-antitoxin mutant biofilm growth for a period of 1 to 4 days. Prior to staining, optical density was read at 600 nm as a measure of overall well growth. Crystal violet-stained biofilm absorbance was read at 550 nm as a measure of biofilm growth. Each graph represents *n* = 4 for each strain except for the MW001 parent, for which *n* = 8. (A) Graph of average OD_600_ measurements of overall well growth ± SEM; (B) graph of average OD_550_ measurements of biofilm growth ± SEM; (C) graph of OD_550_/OD_600_ average measurements of biofilm growth normalized to the overall growth of the well ± SEM; (D) image of crystal violet-stained 4-day biofilms prior to solubilization. Statistically significant difference compared to the MW001 parent within the same day: *, *P* < 0.05; ●, *P* < 0.10. All statistical differences were calculated using a two-way analysis of variance (ANOVA) followed by a Dunnett T3 test.

Using crystal violet staining, the *vapBC14* mutant panel was evaluated for their ability to generate biofilms. Both the Δ*vapC14* toxin and the Δ*vapBC14* toxin-antitoxin mutant were biofilm overproducers compared to the MW001 parent, generating 47% and 65% more biofilm on day 3 and 124% and 119% more biofilm on day 4, respectively (Fig. 3B and D). The Δ*vapB14* antitoxin mutant was deficient in biofilm growth compared to the MW001 parent strain at every time point, with a significant difference on days 1 to 3 (Fig. 3B). Even accounting for the Δ*vapB14* antitoxin mutant’s growth defect by normalizing the biofilm data to the overall growth of the well (optical density at 600 nm [OD_600_]), the Δ*vapB14* antitoxin mutant retained a significant biofilm growth defect on day 2 (47% decrease) and day 3 (37% decrease) (Fig. 3C). The VapB14 antitoxin may act as an activator of biofilm formation by regulating genes such as *abfr1*, which encodes a S. acidocaldarius biofilm repressor, but more evidence is needed to support this possibility. The biofilm defect seen in the Δ*vapB14* antitoxin mutant could be due to the unfettered VapC14 toxin targeting important biofilm RNAs, such as the transcript of the known Lrs14-like biofilm activator (Saci_1223).

### Response of known biofilm genes to absence of the VapB14 antitoxin.

**FIG 4:**
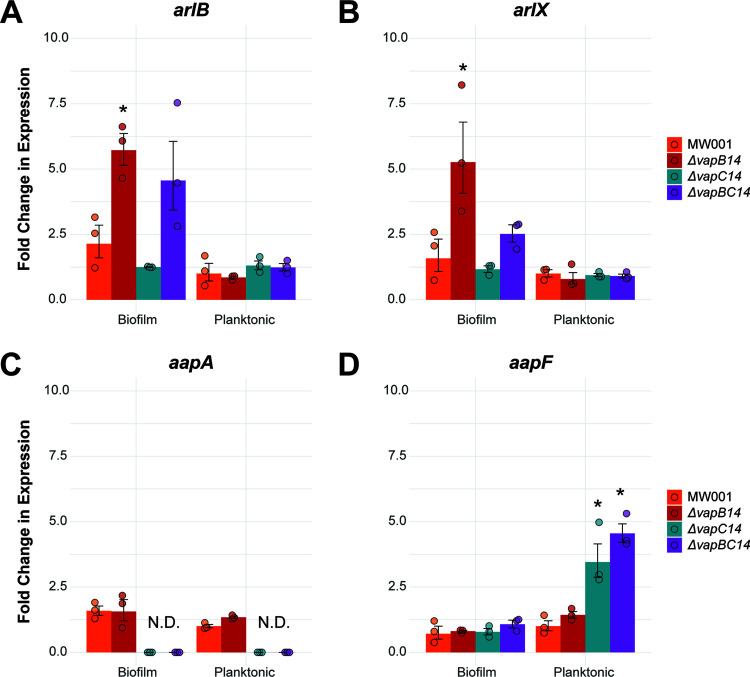
Effect of VapBC14 on the transcriptional expression of the S. acidocaldarius archaeal adhesive pilus and archaellum genes. (A to D) Differential levels of expression of archaellum genes (A) *arlB* and (B) *arlX* and archaeal adhesive pilus genes (C) *aapA* and (D) *aapF* in S. acidocaldarius MW001 parent, Δ*vapB14* antitoxin, Δ*vapC14* toxin, and Δ*vapBC14* antitoxin-toxin mutant biofilm and planktonic cultures. Shown are results from relative qPCR of total RNA isolated from biofilm and planktonic cultures after 3 days of growth. Graphed data represent the average fold change of each gene’s expression of *n* = 3 biological replicates compared to the MW001 parent planktonic condition ± SEM. Graphed points are the fold change in individual biological replicates. Statistically significant difference compared to the MW001 parent under the same condition: *, *P* < 0.05. N.D., none detected as the cycle threshold was above the detection limit. All statistical differences were calculated using a two-way analysis of variance (ANOVA) followed by a Tukey honestly significant difference (HSD) *post hoc* test.

The response of known biofilm genes in the Δ*vapB14* antitoxin mutant was determined via qPCR on 3-day-old biofilm and planktonic samples. The biofilm repressor *abfR1*, Saci_1242 biofilm activator, and UV-inducible pilus genes (*upsE* and *upsA*) registered no response to deletion of the *vapB14* antitoxin gene under any conditions tested (Fig. S4). Strikingly, a 3-fold significant increase was observed in both *arlB* and *arlX* archaellum genes under the Δ*vapB14* antitoxin mutant biofilm condition (Fig. 4A and B). Upregulation of the archaellum in the Δ*vapB14* biofilm, which triggers dispersal from the biofilm, is consistent with the crystal violet experiments showing the Δ*vapB14* mutant produces significantly less biofilm. Furthermore, a slight increase was observed in both archaellum genes in the Δ*vapBC14* double mutant, but no such increase was seen in the Δ*vapC14* single mutant, indicating the importance of the VapB14 antitoxin alone in the regulation of S. acidocaldarius motility. The VapB14 antitoxin directly or indirectly represses the expression of key archaellum genes to minimize biofilm dispersal.

10.1128/mbio.00053-23.4 FIG S4 Differential expression of known biofilm genes in S. acidocaldarius MW001 parent and Δ*vapB14* mutant biofilm and planktonic cultures. Shown are results from relative qPCR of total RNA isolated from S. acidocaldarius MW001 biofilm and planktonic cultures after 3 days of growth. Graphed data represent the average fold change of each gene’s expression of *n* = 3 biological replicates compared to the MW001 parent planktonic condition ± SEM. Graphed points are the fold change in individual biological replicates. Lack of statistical differences was calculated using a two-way analysis of variance (ANOVA) followed by a Tukey honestly significant difference (HSD) *post hoc* test. Download FIG S4, TIF file, 14.4 MB. Copyright © 2023 Lewis et al. 2023 Lewis et al. https://creativecommons.org/licenses/by/4.0/ This content is distributed under the terms of the Creative Commons Attribution 4.0 International license.

Additionally, the deletion of the *vapC14* toxin gene caused transcription of the *aapA* gene to be completely abolished in planktonic and biofilm cells, indicating that VapC14 toxin targets an *aapA* repressor such as AbfR1 (Fig. 4C). However, the binding of AbfR1 is dependent on its phosphorylation state, which differs across these conditions (15); relieving RNase degradation of *abfR1* transcript would not result in complete abrogation of the *aapA* transcription in both planktonic and biofilm cells, as seen here. VapC14 is likely targeting an unknown repressor of *aapA.*

Deletion of the *vapC14* toxin, with or without the presence of the VapB14 antitoxin, caused a significant increase in *aapF* in planktonic cells (3.4-fold in the Δ*vapC14* mutant and 4.4-fold in the Δ*vapBC14* mutant) compared to the MW001 parent (Fig. 4D). Antisense RNA transcripts are known to be within the *Sa. solfataricus aapF* homolog (Sso_2386) (40), and deletion of the *aapF* gene in S. acidocaldarius causes hyperarchaellation (41), which suggests that potential noncoding RNAs within the *aapF* gene may repress archaellum expression. The increase in *aapF* transcription in only Δ*vapC14* toxin mutants indicates that the VapC14 toxin targets either *aapF* mRNA or these antisense transcripts, allowing them to accumulate in its absence. No significant change in *aapF* was measured in the Δ*vapB14* antitoxin mutant under either condition, most likely due to the direct repression of *aapF* by AbfR1 in biofilm cells (14) and the low expression of *vapB14* in planktonic cells (Fig. 1).

### VapBC14 regulation of S. acidocaldarius surface structures.

**FIG 5:**
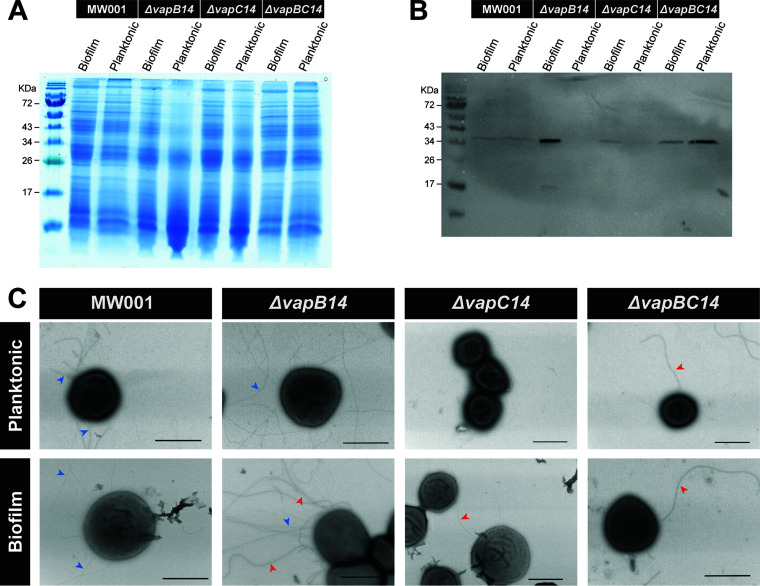
Effect of VapBC14 on S. acidocaldarius archaeal adhesive pilus and archaellum surface structures. (A) Loading control for western blot and (B) archaellum western blot using an anti-ArlB antibody. (C) Transmission electron microscopy images of S. acidocaldarius MW001 parent, Δ*vapB14* antitoxin, Δ*vapC14* toxin, and Δ*vapBC14* antitoxin-toxin mutant biofilm and planktonic cells. Red arrowheads indicate archaella, and blue arrows indicate Aap pili. The scale bar is 1 μm.

Transmission electron microscopy (TEM) of 4-day biofilms showed hyperarchaellation of the Δ*vapB14* antitoxin mutant compared to the MW001 parent and Δ*vapC14* toxin mutant (Fig. 5C). Moreover, an increase in archaella was also detected in the Δ*vapB14* antitoxin mutant biofilm compared to any other strain via western blotting using anti-ArlB antibodies (Fig. 5A and B). EM imaging and western blotting of the Δ*vapBC14* toxin-antitoxin mutant biofilm displayed higher levels of archaella than the MW001 parent (Fig. 5A to C), further confirming the toxin-independent role of the VapB14 antitoxin in the regulation of S. acidocaldarius dispersal. Additionally, both the Δ*vapC14* toxin single mutant and the Δ*vapBC14* double mutant lacked Aap pilus structures, confirming the qPCR results and indicating that the VapC14 toxin is a strong regulator of Aap pilus production. Furthermore, Δ*vapC14* toxin mutant 4-day planktonic cultures were devoid of most surface appendages, supporting the VapC14 toxin’s regulation of Aap pili and archaella in planktonic cells. Thin structures referred to as “threads,” which are structurally similar to type I pili (42), were unaffected by the VapBC14 TA system as they were seen in every strain at similar levels. However, the Δ*vapBC14* double mutant biofilm was hyperpiliated with Ups pili and hyperarchaellated in planktonic culture, which may be stress responses to the loss of other surface appendage structures. (Fig. 5C).

### VapB14 antitoxin homologs across the Sulfolobales and beyond.

**FIG 6:**
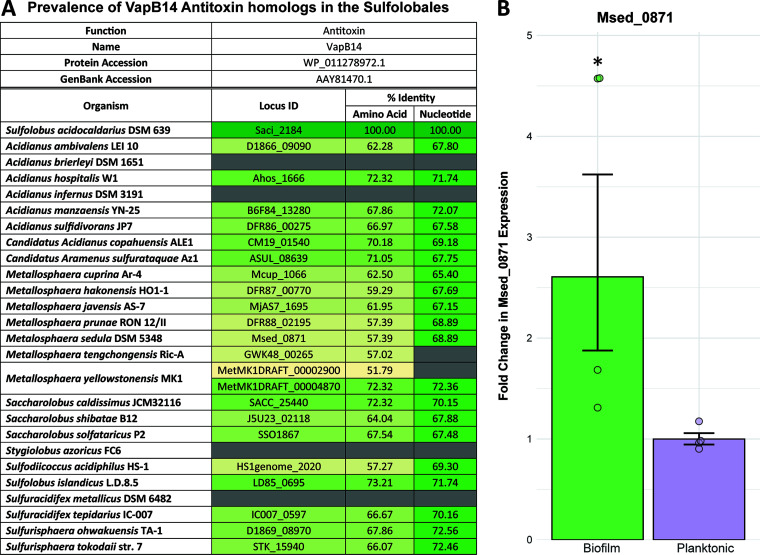
Sulfolobales VapB14 homolog conservation and biofilm response. (A) Prevalence of VapB14 antitoxin homologs in the *Sulfulobales*; (B) differential expression of the Msed_0871 *vapB14* homolog in Metallosphaera sedula biofilm and planktonic cultures. Shown are results from relative qPCR of total RNA isolated from *M. sedula* biofilm and planktonic cultures after 3 days of growth. Graphed data represent the average fold change in Msed_0871 expression of *n* = 4 biological replicates compared to the planktonic condition ± SEM. Graphed points are the fold change in individual biological replicates. Statistically significant difference using a two-tailed Student's *t* test: *, *P* < 0.05.

**FIG 7:**
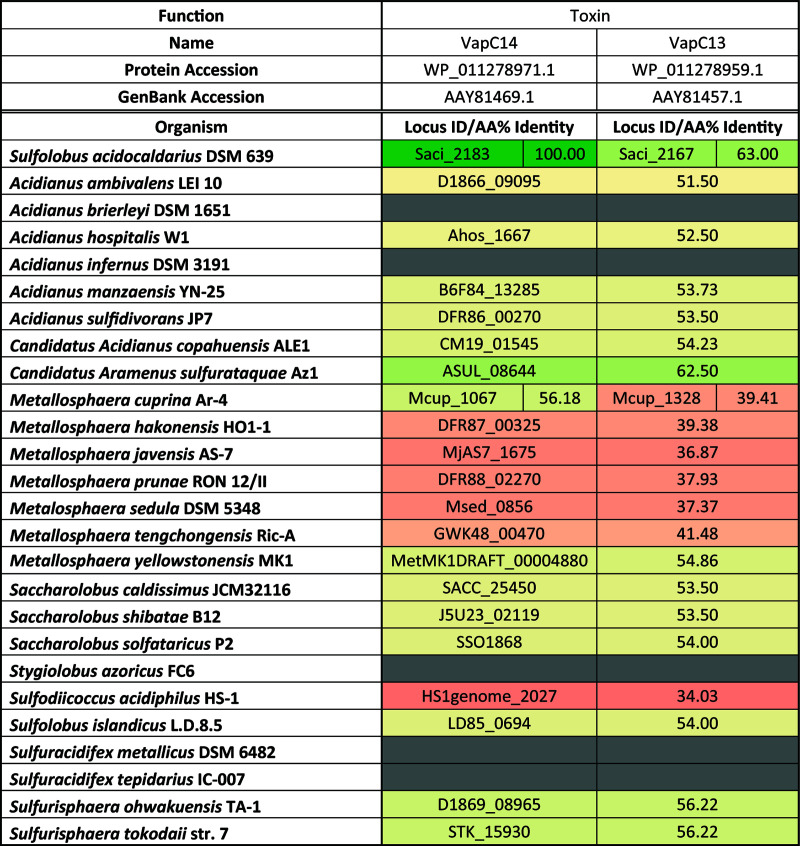
Prevalence of VapC14 toxin homologs in the Sulfolobales.

It was surprising to find that removal of VapB14, comprised of 114 amino acids, had such a profound impact on growth physiology and biofilm formation processes in S. acidocaldarius. This raises the question of whether homologous antitoxins with similar roles exist in other Sulfolobales. In fact, homologs were identified in many Sulfolobales species (Fig. 6A). Interestingly, all surveyed species had at least one homolog of the VapB14 antitoxin, except for Acidianus brierleyi, Acidianus infernus, Stygiolobus azoricus, and Sulfuracidifex metallicus. Metallosphaera yellowstonensis was the only species with two *vapB14* homologs. Conversely, weaker homologs of the VapC14 toxin were found in several Sulfolobales species with a much lower amino acid percentage of identity (Fig. 7). Additionally, synteny analysis using the SyntTax webtool (43) identified several species that contained homologous VapB14 proteins that were not colocalized with the VapC14 toxin gene. Sulfolobales species that have unpaired *vapB14* homologs include several members of the *Metallosphaera* species (Metallosphaera hakonensis, Metallosphaera javensis, Metallosphaera prunae, Metalosphaera sedula, and Metallosphaera tengchongensis), Sulfodiicoccus acidiphilus, and Sulfuracidifex tepidarius. In fact, despite the conservation of a VapB14 homolog (Fig. 6A), a VapC14 homolog is absent from the genome of *Sulfu. tepidarius* (Fig. 7). This suggests that the role of the VapB14 antitoxin in biofilm development may be conserved among the Sulfolobales and less dependent on the conservation of its cognate toxin. To this point, the transcriptional response of the *vapB14* homolog in Metallosphaera sedula, Msed_0871, in 3-day-old biofilms and planktonic cultures was consistent with the response of *vapB14* in *S. acidocaldrius* (Fig. 6B). Since *M. sedula* Msed_0871 has among the lowest homologies found among the Sulfolobales, other *vapB14* homologs with higher similarity likely also play a role in biofilm regulation. Moreover, *M. sedula* does possess both a *vapB14* antitoxin (Fig. 6A) and a *vapC14* toxin homolog (Fig. 7), but they are located at disparate locations in the genome, suggesting divergent functions.

Toxin-antitoxin systems are often associated with mobile genetic elements and are highly susceptible to horizontal gene transfer between species (35). Because of the inherent mobility of these TA systems, it is common to see a large divergence in nucleotide identity even within the same species (35). However, VapB14 homologs across the Sulfolobales have conserved amino acid and nucleotide sequences (Fig. 6A). Furthermore, VapB14 homologs may be pivotal to regulating motility in Archaea as blastp results show VapB14 homologs in many members of the phylum Crenarchaeota and some examples in the Euryarchaeota. Homologs were even identified in motile mesophilic bacterial genera, such as the pathogenic mesophiles of Pseudomonas and marine bacteria of *Nitrosococcus* (see Data Set S1 in the supplemental material). While VapB14 clearly has an important role in the regulation of motility and its homologs are found in many motile prokaryotic species, this antitoxin is also present in many nonmotile genera, such as *Acidianus* of the Sulfolobales, thermophilic bacteria of *Thermoflexus*, and the mesophiles of *Gardneralla* (Data Set S1). VapB14, although important for regulation of the biofilm in the Sulfolobales through controlling dispersal, may have another regulatory role in nonmotile species, such as biofilm attachment.

10.1128/mbio.00053-23.6 DATA SET S1 Sulfolobus acidocaldarius VapB14 blastp results. Download Data Set S1, CSV file, 0.01 MB. Copyright © 2023 Lewis et al. 2023 Lewis et al. https://creativecommons.org/licenses/by/4.0/ This content is distributed under the terms of the Creative Commons Attribution 4.0 International license.

## DISCUSSION

The functional study of toxin-antitoxin systems remains controversial, with recent investigations suggesting no phenotypic response to stressors despite a measured transcriptional response (44). However, this study identifies a VapC14 toxin that significantly impacts RNA transcripts contained in the *aapF* gene and the production of archaella and Aap pilus structures. Additionally, VapC14 RNase activity, although not completely abolished, was significantly reduced by its cognate antitoxin, VapB14, confirming the canonical function of VapB14 as a VapC14-neutralizing antitoxin (Fig. 2B). However, deletion of *vapB14* did not cause the expected upregulation of the *vapC14* toxin gene (Fig. 2C) and may indicate a third-party regulator of the *vapBC14* operon, which has some precedence (45, 46). Saci_1223 is a potential candidate as its deletion results in upregulation of *vapB14* in planktonic cells (Table 2). Deletion of *vapB14* could result in downstream polar effects; however, removal of *vapB14* left no genetic scar, minimizing the potential of this deleterious effect. Furthermore, RNase assays were performed *in vitro* and may not have been representative of the native conditions inside the cell which could lead to stronger affinity of the VapB14 antitoxin for the VapC14 toxin. Also, antitoxins can be promiscuous, meaning that another antitoxin may aid in the reduction of VapC14 RNase activity and that VapB14 may bind a second toxin. The VapC14 toxin’s activity was lower than previously seen for VapC-type toxins in *M. sedula* (34). This lower activity could be due to a higher specificity for a very narrow range of transcripts, such as *aapF*, that may not be well represented in the RNA probes available in the kit used for measuring RNase activity. Overall, RNase activity data demonstrated that VapC14 is an RNase-type toxin and that VapB14 does function as the antitoxin to this activity (Fig. 2B).

The VapC14 toxin most likely activates attachment by targeting a strong repressor of the *aapA* archaeal adhesive pilus structural subunit. This is further supported by the complete lack of Aap pilus structures in the Δ*vapC14* and Δ*vapBC14* mutants (Fig. 5C). However, these mutants also are biofilm overproducers, which suggests they are employing an alternative attachment mechanism. Threads, the only non-type IV pilus surface filament on S. acidocaldarius, are present in EM images of all strains. Although the function of threads is still unknown, S. acidocaldarius Ups pilus, Aap pilus, and archaellum triple mutants are capable of making biofilm (6). Threads may be playing a compensatory attachment role in the Δ*vapC14* and Δ*vapBC14* mutants, allowing these strains to produce biofilm. Similarly, the Ups hyperpiliation of the Δ*vapBC14* double mutant may also improve its biofilm production (Fig. 5C). The Δ*vapBC14* double mutant biofilm also had an increase in archaella compared to the parent, which could contribute to biofilm formation as archaella can aid in initial attachment (6, 47). Finally, S. acidocaldarius *aapX* and *aapE* mutants produce more extracellular matrix, which would contribute to biofilm formation (41). As there were no visible Aap pili in the Δ*vapC14* single or Δ*vapBC14* double mutant (Fig. 5C), it is reasonable to assume that AapX and AapE are absent in these strains yielding excess extracellular matrix. Overall, Δ*vapC14* toxin and Δ*vapBC14* double mutants may be biofilm overproducers through alternative attachment mechanisms and overexpression of extracellular matrix.

**FIG 8:**
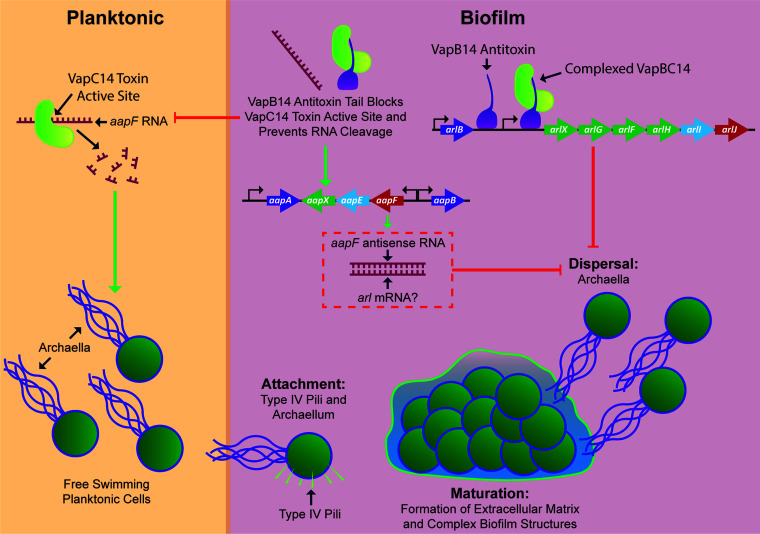
Archaellum regulation by the VapB14 antitoxin and VapC14 toxin. Under planktonic conditions, the VapC14 toxin degrades *aapF* RNA transcripts, relieving archaella repression and inducing motility. Under biofilm conditions, the VapB14 antitoxin functions by both transcriptionally repressing the archaellum (*arl*) locus and suppressing the posttranscriptional degradation of the *aapF* transcript by the VapC14 toxin, leading to a downregulation in motility and induction of biofilm formation.

Additionally, in planktonic cells, *aapF* is significantly increased in the absence of the VapC14 toxin (Fig. 4D), which may contain antisense noncoding RNAs like those observed in the *Sa. solfataricus aapF* homolog (40). Furthermore, deletion of *aapF* in S. acidocaldarius causes an increase in archaella (41), suggesting that AapF or potential noncoding transcripts within the *aapF* gene repress archaella expression. An abundance of antisense *aapF* transcript may function as noncoding RNAs that posttranscriptionally downregulate archaellum gene expression. As is natural for a toxin-antitoxin system, VapC14 and VapB14 may apply opposing regulatory pressure on archaellum expression. VapC14 derepresses the archaella by degrading *aapF* mRNA or a noncoding RNA during planktonic growth. However, during biofilm growth VapB14 is highly expressed and transcriptionally represses the archaellum. VapB14 also behaves as a traditional antitoxin by neutralizing the VapC14 toxin, allowing the archaellum to be posttranscriptionally repressed (Fig. 8). While nutrient starvation is known to induce expression of the archaellum in S. acidocaldarius (48) through regulation by ArnR (12), the VapBC14 TA system’s regulation of the archaellum is responsive to biofilm growth rather than nutrient availability.

Unlike Bacteria, noncoding RNAs are plentiful in the genomes of Archaea (40). Since Archaea lack sigma factors and have an abundance of type II toxin-antitoxin systems, this may point to RNase activity of type II toxins as an important regulatory mechanism within this domain. Specifically, homologs of both AapF and VapB14 are found in most surveyed Sulfolobales species (Fig. 6A; see Fig. S5, Data Set S3, and Text S1 in the supplemental material), suggesting that the mechanism of archaellum regulation described here may be a multispecies phenomenon that is present in all archaellated Sulfolobales. This is further supported by the similar upregulation of the *M. sedula* VapB14 homolog (Msed_0871) to biofilm growth (Fig. 6B). Additionally, conservation of *vapB14* is independent of *vapC14*, as several Sulfolobales species either carry these genes at distinct locations or possess only a *vapB14* antitoxin gene. Moreover, VapB14 homologs are prevalent in the archaeal phylum Crenarchaeota and present in some species of the phylum Euryarchaeota (see Data Set S1 in the supplemental material). While the importance of VapB14 in motile Archaea is evident, homologs are also found in some bacterial species and nonmotile organisms, indicating that VapB14 may also have other functions.

10.1128/mbio.00053-23.5 FIG S5 Prevalence of archaellum and type IV pilus homologs in the Sulfolobales. Download FIG S5, TIF file, 14.4 MB. Copyright © 2023 Lewis et al. 2023 Lewis et al. https://creativecommons.org/licenses/by/4.0/ This content is distributed under the terms of the Creative Commons Attribution 4.0 International license.

10.1128/mbio.00053-23.9 TEXT S1 Supplemental results and methods. Download TEXT S1, DOCX file, 0.04 MB. Copyright © 2023 Lewis et al. 2023 Lewis et al. https://creativecommons.org/licenses/by/4.0/ This content is distributed under the terms of the Creative Commons Attribution 4.0 International license.

TA systems are prevalent in bacteria, archaea, and fungi (16), suggesting that the evolution of the current type II TA systems is not a recent occurrence. Fossil evidence has also shown that prokaryotic organisms of both bacterial and archaeal origins were forming multicellular biofilms more than 3 billion years ago (1). In fact, the earliest recorded occurrences of biofilms are in hydrothermal environments like those native to the species of the Sulfolobales (1). It is, therefore, possible that this VapBC14 TA system may have coevolved with the ability to form a biofilm within this thermophilic order. Furthermore, nucleotide sequence conservation of a type II TA system is atypical due to their association with mobile genetic elements and tendency toward horizontal gene transfer (35, 49). However, VapB14 is highly conserved across the Sulfolobales (Fig. 6A), indicating an evolutionary selective pressure to maintain this small but important biofilm-regulating protein. Overall, the bifunctional VapB14 antitoxin has evolved as an important regulator of Sulfolobales, and perhaps archaeal, motility not only by inhibiting the activity of its cognate toxin but also through transcriptional repression of the archaellum (Fig. 8).

## MATERIALS AND METHODS

### RNase activity assay.

RNase activity assays were performed as described previously using the RNaseAlert kit (Integrated DNA Technologies) (34). For each reaction mixture containing VapC14, 5 μg of VapC14 toxin was added, with or without the addition of 10, 20, or 30 μg of VapB14 antitoxin or 25 mM EDTA. A no-protein control and 10 μg of a VapB14 antitoxin-alone control were also performed. All reaction mixtures were prewarmed at 75°C for 5 min to activate the VapC14 toxin and VapB14 antitoxin.

### Planktonic growth curves.

Cultures were inoculated from frozen stocks in 50 mL 75°C prewarmed Brock’s salts (pH 3) supplemented with 0.1% NZ-amine, 0.2% sucrose, and 0.01g/L uracil. Cultures of S. acidocaldarius MW001 and the Δ*vapB14*, Δ*vapC14*, and Δ*vapBC14* mutants were grown aerobically in foam-stoppered flasks at a 1:5 volume/flask ratio at 75°C at 150 rpm. Cultures were monitored by back scatter at 520 nm every 15 min with the Cell Growth Quantifier (Scientific Bioprocessing, Inc.) for 72 h.

### Crystal violet biofilm assay.

The S. acidocaldarius MW001 parent and Δ*vapB14* antitoxin, Δ*vapC14* toxin, and Δ*vapBC14* toxin-antitoxin mutant biofilms were grown in 1 mL of Brock’s basal salts (pH 3) with 0.1% NZ-amine, 0.2% sucrose, and 0.01 g/L uracil on Sarstedt Cell^+^ flat-bottom 24-well plates at 75°C for a period of 1 to 4 days. The outer wells of each 24-well plate were filled with 1 mL of water, and plates were incubated in a humidified box to reduce evaporation. Prior to staining, optical density at 600 nm (OD_600_) was read as a measure of overall well growth. Supernatant was then removed, attached biofilm was stained with 500 μL 0.1% crystal violet, the biofilm was washed twice with 1 mL of water, crystal violet was solubilized with 500 μL 100% ethanol, and absorbance was read at 550 nm.

### RNA isolation and quantitative real-time PCR.

S. acidocaldarius MW001 and mutant planktonic cultures were grown in 50 mL 75°C prewarmed Brock’s salts (pH 3) supplemented with 0.1% NZ-amine, 0.2% sucrose, and 0.01g/L uracil. Cultures were inoculated at an OD_600_ of 0.01 and grown aerobically in foam-stoppered flasks at a 1:5 volume/flask ratio at 75°C at 150 rpm for 4 days. On day 2, 20 mL of sterile 75°C deionized water was added to each flask. The entire culture of cells was centrifuged at 4,000 × *g* for 5 min and resuspended in 1 mL of RNAlater (Invitrogen). S. acidocaldarius MW001 and mutant biofilm cultures were inoculated in the same prewarmed medium at an OD_600_ of 0.01 in 150- by 20-mm Sarstedt Cell^+^ tissue culture dishes (Sarstedt). Biofilms were incubated at 75°C in a sealed humidified box for 4 days. RNA was then extracted on days 1 to 4 for S. acidocaldarius MW001 and the Δ*vapB14* antitoxin mutant and on day 3 for the Δ*vapC14* toxin mutant and Δ*vapBC14* double mutant using TRIzol reagent followed by the RNeasy kit (Qiagen) (50). Residual DNA was removed by a rigorous treatment with Turbo DNase (Invitrogen). RNA was determined to be relatively free of DNA contamination by qPCR, checking for amplification using *secY* primers (see Data Set S2 in the supplemental material) with RNA as the template. Relative qPCR was performed using SsoFast EvaGreen supermix (Bio-Rad) or SsoAdvanced Universal SYBR green supermix (Bio-Rad), and fold change values were calculated using the Livak method (51) with *secY* used as the normalizer.

10.1128/mbio.00053-23.7 DATA SET S2 Primer, plasmid, and strain tables. Download Data Set S2, XLSX file, 0.02 MB. Copyright © 2023 Lewis et al. 2023 Lewis et al. https://creativecommons.org/licenses/by/4.0/ This content is distributed under the terms of the Creative Commons Attribution 4.0 International license.

*M. sedula* planktonic and biofilm cultures were inoculated as described above with the following exceptions. Cultures were inoculated in 50 mL 70°C prewarmed Brock’s salts (pH 2) supplemented with 0.1% yeast extract and incubated at 70°C at 150 rpm for planktonic cultures and 70°C stationary for biofilm plates for 3 days. RNA was determined to be relatively free of DNA contamination by qPCR, checking for amplification using Msed_R0026 16s gene primers (Data Set S2) with RNA as the template. Relative qPCR was performed using SsoAdvanced Universal SYBR green supermix (Bio-Rad), and fold change values were calculated using the Livak method (51) with Msed_R0026 used as the normalizer.

### Transmission electron microscopy of S. acidocaldarius surface appendage structures.

Biofilms and planktonic S. acidocaldarius MW001 and mutant strains were grown as for RNA isolation. Biofilm was scraped off the petri dishes and resuspended in 1 mL growth medium. Five microliters of biofilm or planktonic cells were applied on freshly glow-discharged carbon/Formvar-coated copper grids (300 mesh; Plano GmbH) and incubated for 30 s. The excess liquid was blotted away, and cells were negatively stained with 2% uranyl acetate. Imaging was done with Hitachi HT7800 operated at 100 kV, equipped with an EMSIS Xarosa 20-megapixel CMOS camera.

### Western blotting.

To assay the production of ArlB in planktonic cells and biofilm, S. acidocaldarius MW001 and mutant strains were grown as for RNA isolation. Biofilm was scraped off the petri dishes and resuspended in 1 mL growth medium. The OD_600_s of cells from biofilm and planktonic cultures were determined. Cells were pelleted at 2,400 × *g* in a tabletop centrifuge for 10 min and resuspended to a theoretical OD of 10 in 1× SDS loading dye. The whole-cell samples were separated on SDS-PAGE and blotted on polyvinylidene difluoride (PVDF) membrane (Roche). The membrane was blocked with I-Block (Thermo Fisher Scientific) and incubated in primary antibody against ArlB (Eurogentec) overnight at 4°C. Afterwards, the membrane was incubated in secondary goat anti-rabbit antibody coupled to horseradish peroxidase (HRP) overnight at 4°C. Chemiluminescent signals were recorded with IBright 1500 (Invitrogen, Thermo Fisher Scientific) using Clarity Western ECL enhanced chemiluminescence blotting substrate (Bio-Rad).

### Data availability.

Microarray data are available at the Gene Expression Omnibus repository (NCBI) under accession no. GSE226483 for normalized data and accession no. GSM7077821, GSM7077822, GSM7077823, and GSM7077824 for raw data files. See Text S1 for additional methods.

10.1128/mbio.00053-23.8 DATA SET S3 Sulfolobales biofilm gene homologs and pangenome. Download Data Set S3, XLSX file, 5.0 MB. Copyright © 2023 Lewis et al. 2023 Lewis et al. https://creativecommons.org/licenses/by/4.0/ This content is distributed under the terms of the Creative Commons Attribution 4.0 International license.
