# Supplementary figures and images for: Stay or Go: Sulfolobales Biofilm Dispersal Is Dependent on a Bifunctional VapB Antitoxin
Source: mBio. 2023 Apr 10;14(2):e00053-23. doi: 10.1128/mbio.00053-23 (PMC10127717; doi:10.1128/mbio.00053-23)

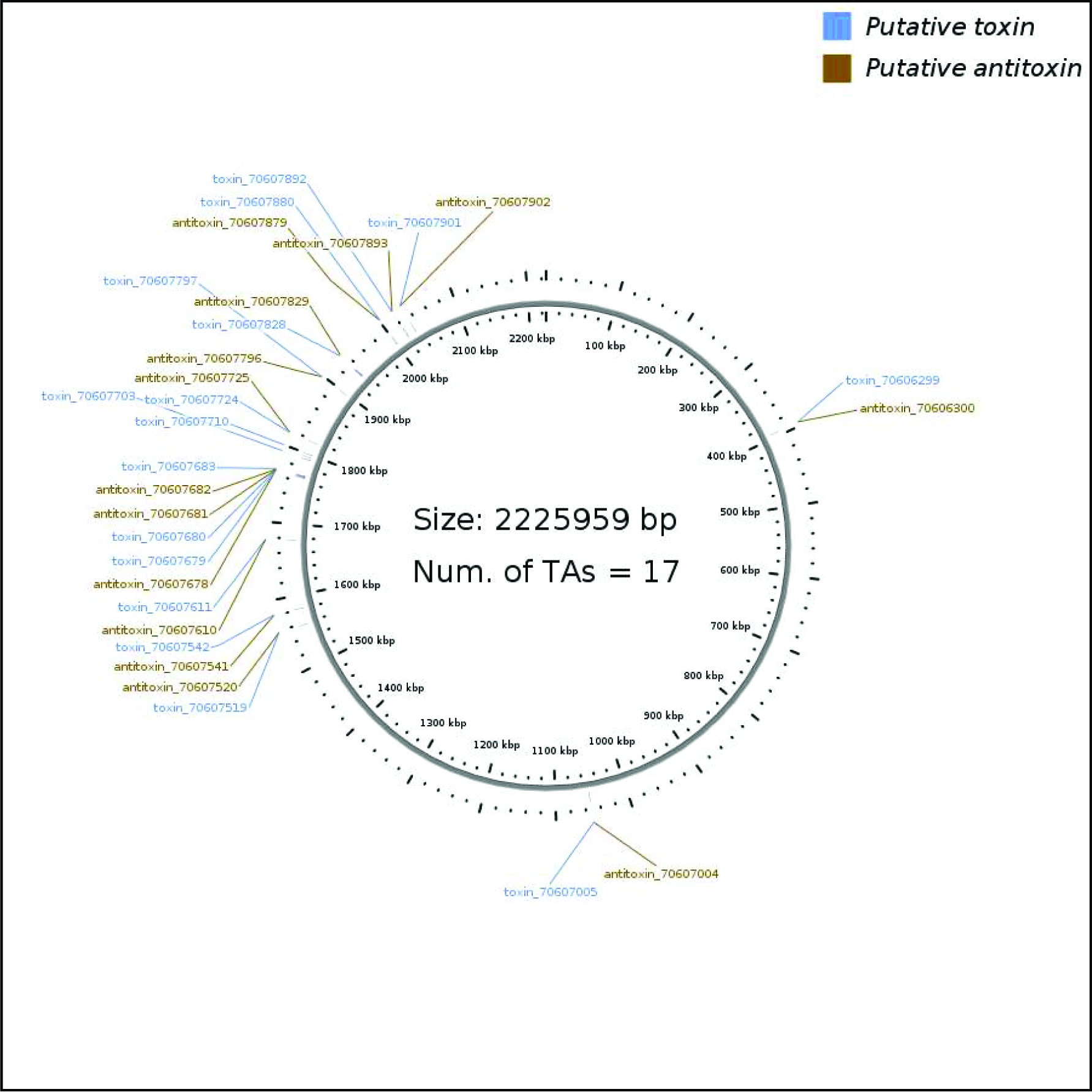

Supplement: FIG S1 [file mbio.00053-23-s0001.tif]

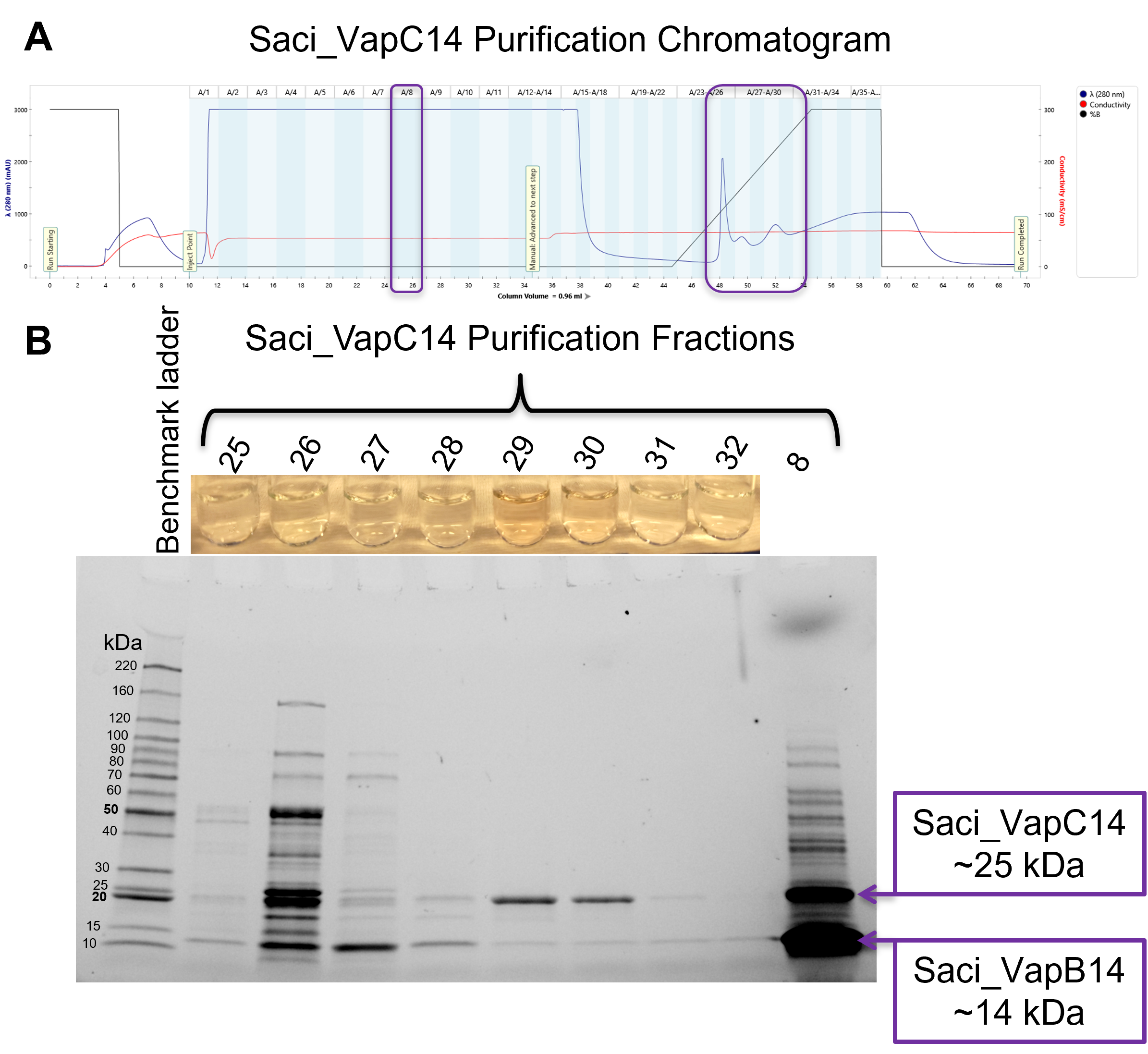

Supplement: FIG S2 [file mbio.00053-23-s0002.tif]

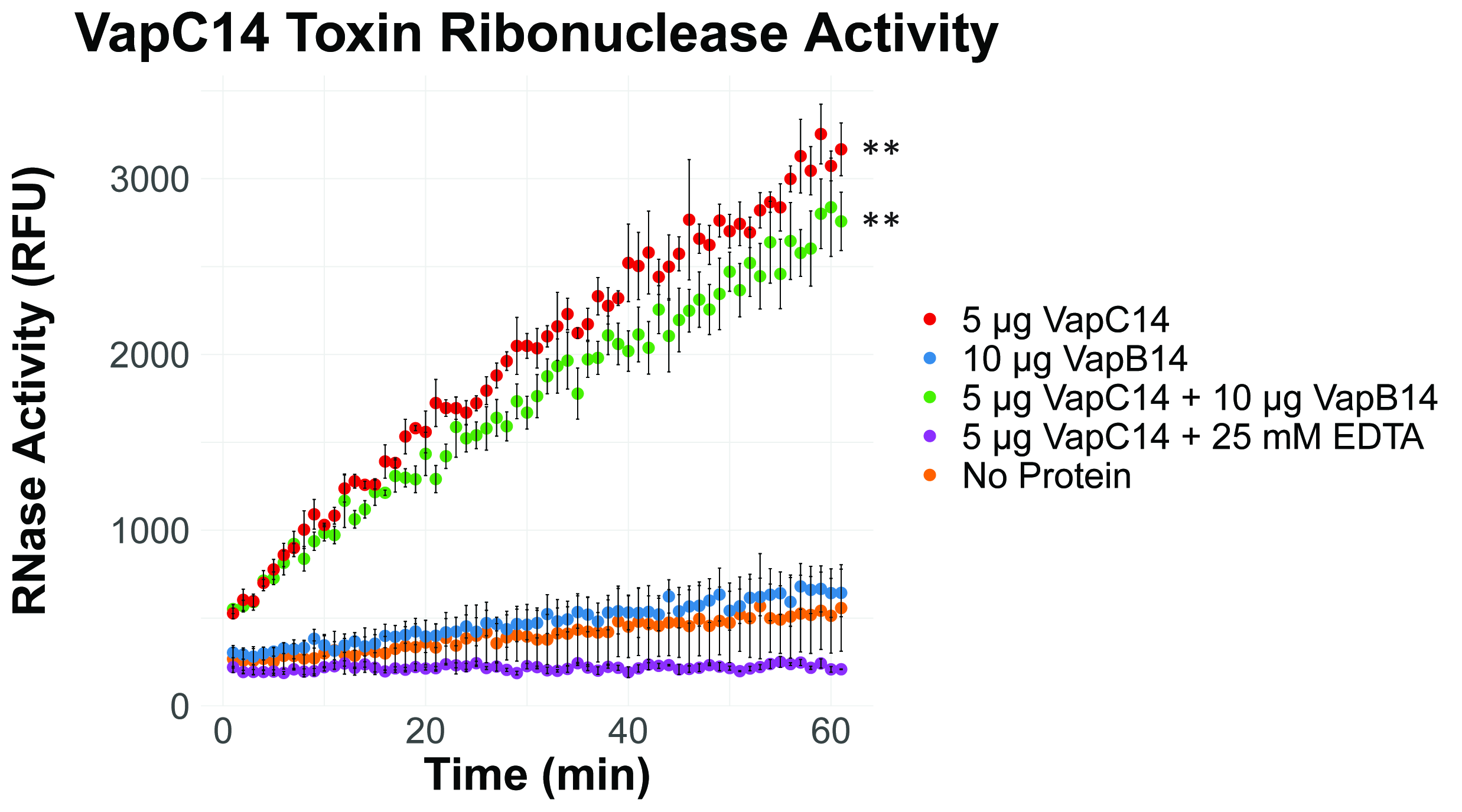

Supplement: FIG S3 [file mbio.00053-23-s0003.tif]

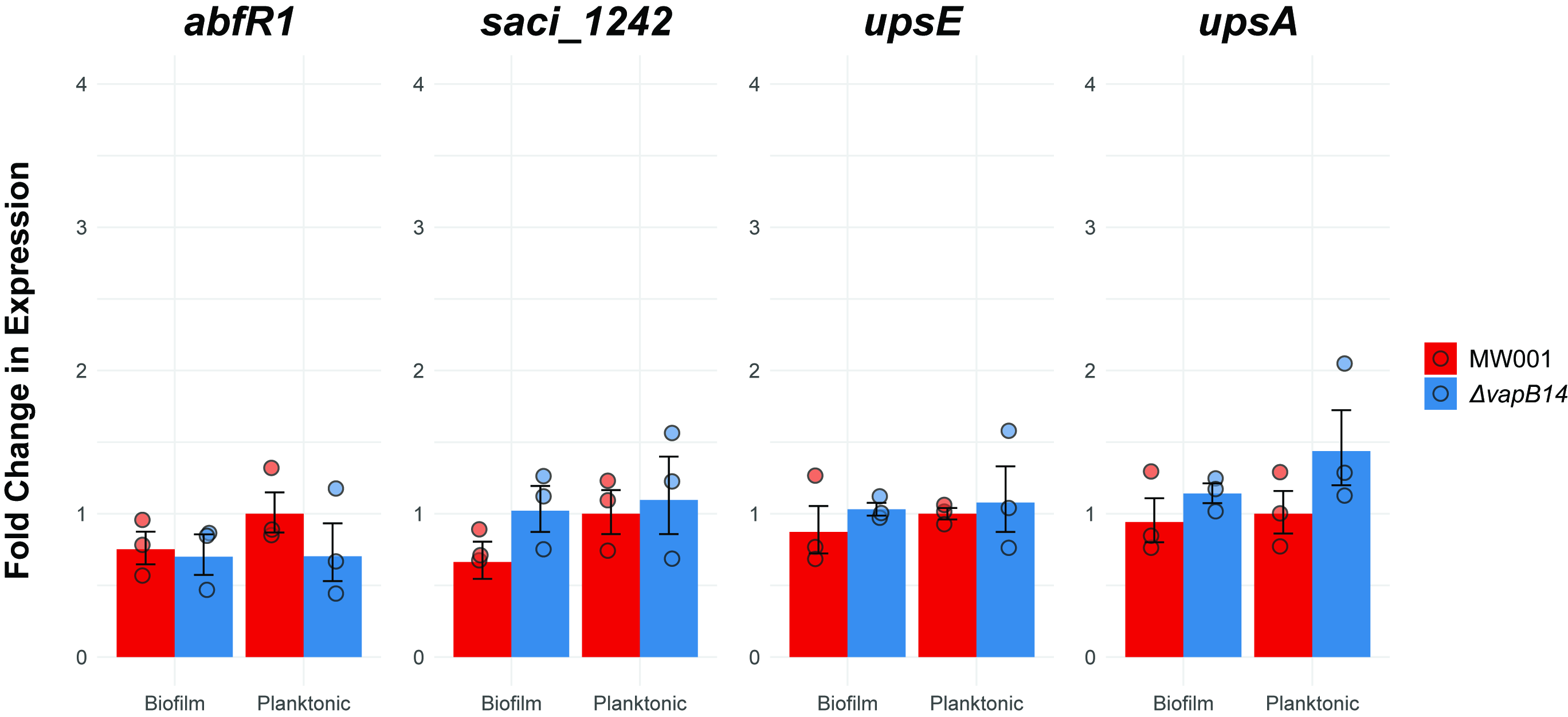

Supplement: FIG S4 [file mbio.00053-23-s0004.tif]

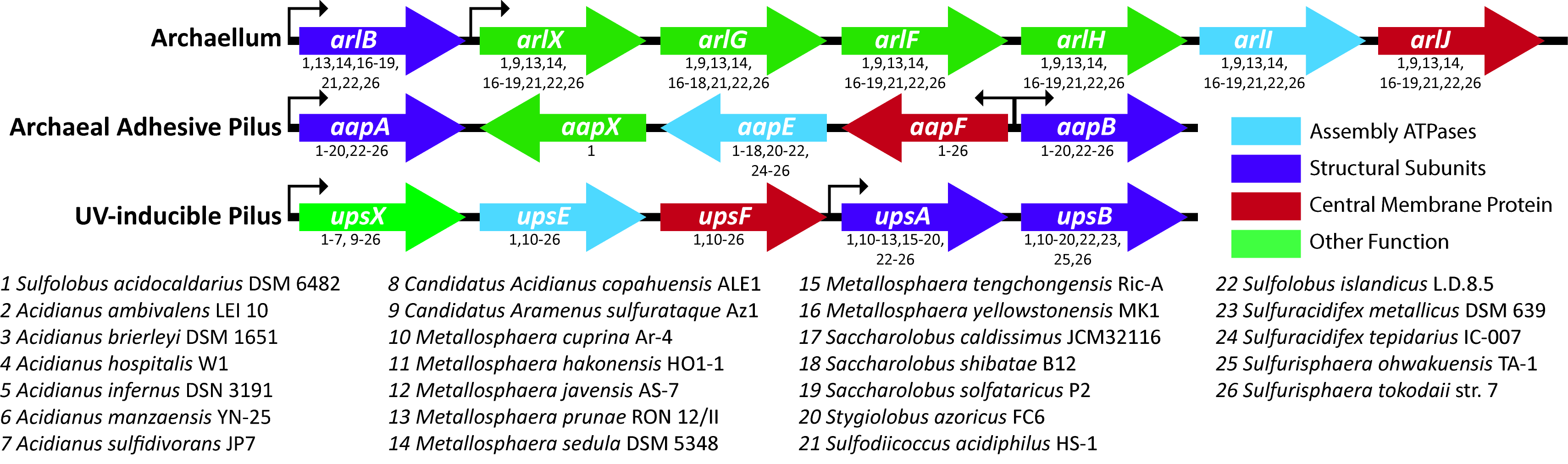

Supplement: FIG S5 [file mbio.00053-23-s0005.tif]
