# Supplementary material for: Stay or Go: Sulfolobales Biofilm Dispersal Is Dependent on a Bifunctional VapB Antitoxin
Source: mBio. 2023 Apr 10;14(2):e00053-23. doi: 10.1128/mbio.00053-23 (PMC10127717; doi:10.1128/mbio.00053-23)
Supplement: TEXT S1 [file mbio.00053-23-s0009.docx]

**Stay or Go: Sulfolobales Biofilm Dispersal is Dependent on a Bifunctional VapB Antitoxin**

April M. Lewis, Daniel J. Willard, Mohamad J.H. Manesh, Shamphavi Sivabalasarma, Sonja-Verena Albers, and Robert M. Kelly

# Supplemental Results

## Biofilm-related genes across the Sulfolobales

Among the Sulfolobales, the biofilm structure and function of *Sulfolobus acidocaldarius* is the most studied (1-3)*.* Using *S. acidocaldarius* as the basis, homologs of known biofilm genes and regulators could be identified in 26 sequenced species within the Sulfolobales (**Fig. S5, Data Set S3**). As expected, genes encoding the archaellum were present in many species but absent in non-motile archaea, such as *Acidianus* species. Additionally, *Candidatus Acidianus copahuensis*, *Metallosphaera cuprina*, *Metallosphaera hakonensis*, and *Metallosphaera javensis* are lacking archaellum genes, suggesting that these species are also non-motile. The *arlH* and *arlI* archaellum genes, which function in archaellum assembly and rotation, are highly conserved across the Sulfolobales (**Fig. S5, Data Set S3**). The *arlB* archaellum structural subunit is also highly conserved (58-70% amino acid sequence identity). Interestingly, nearly all sequenced species have the key genes needed to create Aap pili, except for *Sulfodiicoccus acidiphilus*, which is missing the structural subunits, and *Sulfuracidifex metallicus* that is missing the assembly ATPase. Although not identified in this bioinformatic analysis, *Saccharolobus solfataricus* has an Aap pili ATPase (Sso_2387) that has been experimentally characterized and known to autophosphorylate (4). Notably, the Aap pili gene, *aapX*, is found only in *S. acidiocaldarius.* Though absent in other species, *aapX* is vital for pilus production in *S. acidocaldarius* (5). In fact, deletion of any single *aap* gene in *S. acidocaldarius* yields cells with no Aap pili structure (5). These species may have lost the ability to make this pilus structure and identified genes are an artifact of that lost ability. Considering that the homology of these Aap genes is low in most cases, species containing homologs may be making similar but altered structures. The one caveat is the *aapE* assembly ATPase gene, which is highly conserved. Furthermore, all *Acidianus* species, as well as *Candidatus Acidianus copahuensis*, *Candidatus Aramenus sulfurataque*, and *Sulfodiicoccus acidphilus* are missing major components of the UV-inducible pilus. Species with homologs to most of the UV-inducible genes have low homology (**Data Set S3**) and may not closely resemble the structure in *S. acidocaldarius*.

Homologs of known *S. acidocaldarius* biofilm activators, encoded by *arnR* (Saci_1180), *arnR1* (Saci_1171), Saci_1223 and Saci_1242, and the known biofilm repressor AbfR1 (Saci_0446,) could be identified in sequenced Sulfolobales species (**Data Set S3**). The Lrs14-like protein product of Saci_1223 has no homologs in any other Sulfolobales species. Additionally, both ArnR and ArnR1, homologs of each other, only have homologs in a few species, albeit with low sequence identity. On the other hand, the biofilm repressor gene, *abfR1*, is highly conserved in every species, except for *Sulfod. acidiphilus.* Similarly, the biofilm activator gene Saci_1242 also has homologs in all Sulfolobales species, except for *M. cuprina.* The conservation of these biofilm regulators indicates that they serve a similar function in biofilm dynamics of these other Sulfolobales.

# Supplemental Materials and Methods

## Generation of S. acidocaldarius Toxin and Antitoxin mutants

Generation of the *ΔvapC14* Toxin and the *ΔvapB14* Antitoxin single mutants were done be amplifying the flanking regions of each gene from *S. acidocaldarius* MW001 genomic DNA with complementary overhangs and using Gibson assembly (NEBuilder Hifi Master Mix, New England Biolabs) to clone the flanking regions into the pSVA406 suicide vector that contains the *pyrEF* uracil genes. To create the *ΔvapBC14* Toxin-Antitoxin double mutant, primers were used to amplify the pSVA406 backbone and the flanking regions of the *vapBC14* operon excluding the *vapB14* gene off the p*ΔvapB14* plasmid. This linear product was then circularized with a kinase, ligase, DpnI (KLD, New England Biolabs) reaction to generate the p*ΔvapBC14* double mutant suicide vector. All constructed plasmids were transformed into the *Escherichia coli* cloning strain NEB5α (New England Biolabs) according to the manufacturer’s instructions (See **Data Set S2)**.

Each mutant suicide vector was used in a previously described stepwise cloning method (6) to generate the associated mutant in the *S. acidocaldarius* MW001 uracil auxotroph strain. Briefly, each mutant plasmid was electroporated into the *S. acidocaldarius* MW001 strain and plasmid integration was selected for on 1^st^ selection plates without uracil. Using colony PCR, clones were confirmed to have integrated plasmid and were sub-cultured into nonselective liquid medium containing uracil. Liquid cultures were grown for 2-3 days statically at 75°C, serial diluted, and plated on 2^nd^ selection plates containing both uracil and the 5-FOA counterselection. Liquid cultures were also passaged twice into fresh nonselective medium, grown for an additional 2-3 days, and plated on 2^nd^ selection plates. Mutant generation was confirmed with colony PCR and sequencing (See **Data Set S2)**.

## Generation of VapB14 and VapBC14 expression strains

Codon optimized sequences of the *vapC14* Toxin gene and *vapB14* Antitoxin gene were generated by Genewiz and cloned separately into the pET46 expression vector (Novagen) using restriction cloning so that each protein was N-terminally His-tagged on their own plasmid. The resulting pET46_VapB14 plasmid was then transformed into the Rosetta 2(DE3) expression strain (MilliporeSigma) following the manufacturer’s instructions. Using the pET46_VapB14 and pET46_VapC14 plasmids as template the codon optimized *vapB14* Antitoxin and *vapC14* Toxin genes were PCR amplified with complementary Gibson overhangs for the pETDuet-1 co-expression vector (Novagen). The pETDuet-1 backbone and intergenic region were then PCR amplified with complementary Gibson overhangs for either the *vapC14* or *vapB14* codon optimized genes. Using Gibson assembly (NEBuilder Hifi Master Mix, New England Biolabs) the pETDuet_VapBC14 was generated with the VapC14 Toxin N-terminally His-tagged and the VapB14 Antitoxin untagged. The resulting pETDuet_VapBC14 plasmid was then transformed into the Rosetta 2(DE3) pLysS expression strain (MilliporeSigma) following the manufacturer’s instructions. All constructed plasmids were transformed into the *E. coli* cloning strain NEB5α (New England Biolabs) according to the manufacturer’s instructions (See **Data Set S2)**.

## Expression of the VapB14 Antitoxin and the VapC14 Toxin

Overnight cultures of the VapB14 Antitoxin expression strain and the VapBC14 co-expression strain were inoculated at a 1% (v/v) in LB and incubated at 37°C, 250 rpm. Once cultures reached an OD_600_ ~ 1, expression was induced with the addition of 1 mM Isopropyl β-d-1-thiogalactopyranoside (IPTG) and incubate at 20°C, 250 rpm for 18 hrs. Cells were then pelleted by centrifugation, resuspended in immobilized metal affinity chromatography (IMAC) binding buffer (50 mM sodium phosphate, 300 mM sodium chloride, 20 mM imidazole, pH 8.0), and lysed using a French Pressure Cell (SLM-AMINCO) at a pressure of 16000 psi. Lysate was then heat treated at 65°C for 20 min, centrifuged at 12000xg for 30 min, and then sterile filtered. Clarified cell lysate was then loaded onto a HisTrap 1 mL column (GE) and a linear gradient of elution buffer (50 mM sodium phosphate, 300 mM sodium chloride, 500 mM imidazole, pH 8.0) was applied to the column to elute bound his-tagged protein. Fractions were visualized on a 4-20% SDS-PAGE gel (BioRad). The VapB14 Antitoxin readily dissociated from the His-tagged VapC14 Toxin in the co-expression lysate and no additional steps were required to obtain the VapC14 Toxin alone. Fractions with purified protein of interest were combined and dialyzed into RNase activity buffer (10 mM MgCl_2_, 150 mM NaCl, 50 mM Tris-HCL at pH 6.0). Protein was then quantified using a Bradford assay and a Biotek plate reader.

## Generation of the Sulfolobales protein database

Genomes for the species investigated in this study are available in the NCBI database and were used to identify protein homology of known biofilm structures and regulators between Sulfolobales species. Accession numbers for the genomes can be found in **Data Set S3** Protein homology for whole genomes were evaluated using the GET_HOMOLOGUES suite (7, 8) with default settings for the orthoMCL algorithm (9) BLASTP and allowing for clusters containing only a single protein sequence.

## DNA microarray analysis of S. acidocaldarius planktonic and biofilm cells.

Biofilms and planktonic *S. acidocaldarius* MW001 and ΔSaci_1223 cultures were grown as previously described (10). A whole-genome oligonucleotide microarray for *S. acidocaldarius* MW001 was developed based on the reported genome sequence and fabricated, as reported previously (11), but with a few modifications. Microarray slides were printed with four replicates per probe spotted onto each array to strengthen statistical analysis. From each sample RNA was extracted using the Qiagen RNAqueous kit (Qiagen, Valencia, CA), following the manufacturer’s instructions. Equal amounts of RNA from each biological replicate were pooled and cDNA synthesized. Samples were then hybridized, slides were scanned, and data analyzes as previously described (12).

# References:

1. Schult F, Le TN, Albersmeier A, Rauch B, Blumenkamp P, van der Does C, Goesmann A, Kalinowski J, Albers S-V, Siebers B. 2018. Effect of UV irradiation on *Sulfolobus acidocaldarius* and involvement of the general transcription factor TFB3 in the early UV response. Nucleic Acids Res 46:7179-7192.

2. Henche A-L, Koerdt A, Ghosh A, Albers S-V. 2012. Influence of cell surface structures on crenarchaeal biofilm formation using a thermostable green fluorescent protein. Environ Microbiol 14:779-793.

3. Jarrell KF, Albers S-V. 2012. The archaellum: an old motility structure with a new name. Trends in Microbiol 20:307-312.

4. Albers S-V, Driessen AJ. 2005. Analysis of ATPases of putative secretion operons in the thermoacidophilic archaeon *Sulfolobus solfataricus*. Microbiology 151:763-773.

5. Henche AL, Ghosh A, Yu X, Jeske T, Egelman E, Albers SV. 2012. Structure and function of the adhesive type IV pilus of *Sulfolobus acidocaldarius*. Environ Microbiol 14:3188-3202.

6. Wagner M, van Wolferen M, Wagner A, Lassak K, Meyer BH, Reimann J, Albers S-V. 2012. Versatile genetic tool box for the crenarchaeote *Sulfolobus acidocaldarius*. Front Microbiol 3:214-214.

7. Contreras-Moreira B, Cantalapiedra CP, Garcia-Pereira MJ, Gordon SP, Vogel JP, Igartua E, Casas AM, Vinuesa P. 2017. Analysis of Plant Pan-Genomes and Transcriptomes with GET_HOMOLOGUES-EST, a Clustering Solution for Sequences of the Same Species. Front Plant Sci 8:184.

8. Contreras-Moreira B, Vinuesa P. 2013. GET_HOMOLOGUES, a versatile software package for scalable and robust microbial pangenome analysis. Appl Environ Microbiol 79:7696-701.

9. Li L, Stoeckert CJ, Jr., Roos DS. 2003. OrthoMCL: identification of ortholog groups for eukaryotic genomes. Genome Res 13:2178-89.

10. Koerdt A, Orell A, Pham TK, Mukherjee J, Wlodkowski A, Karunakaran E, Biggs CA, Wright PC, Albers S-V. 2011. Macromolecular Fingerprinting of *Sulfolobus* Species in Biofilm: A Transcriptomic and Proteomic Approach Combined with Spectroscopic Analysis. J Proteome Res 10:4105-4119.

11. Tachdjian S, Kelly RM. 2006. Dynamic metabolic adjustments and genome plasticity are implicated in the heat shock response of the extremely thermoacidophilic archaeon *Sulfolobus solfataricus*. J Bacteriol 188:4553-4559.

12. Cooper CR, Lewis AM, Notey JS, Mukherjee A, Willard DJ, Blum PH, Kelly RM. 2023. Interplay between transcriptional regulators and VapBC toxin‐antitoxin loci during thermal stress response in extremely thermoacidophilic archaea. Environ Microbiol.
